# An Overview of Potential Oleaginous Microorganisms and Their Role in Biodiesel and Omega-3 Fatty Acid-Based Industries

**DOI:** 10.3390/microorganisms8030434

**Published:** 2020-03-19

**Authors:** Alok Patel, Dimitra Karageorgou, Emma Rova, Petros Katapodis, Ulrika Rova, Paul Christakopoulos, Leonidas Matsakas

**Affiliations:** 1 Biochemical Process Engineering, Division of Chemical Engineering, Department of Civil, Environmental, and Natural Resources Engineering, Luleå University of Technology, SE-971 87 Luleå, Sweden; alok.kumar.patel@ltu.se (A.P.); emmaarova@hotmail.com (E.R.); ulrika.rova@ltu.se (U.R.); paul.christakopoulos@ltu.se (P.C.); 2 Laboratory of Biotechnology, Department of Biological Applications and Technologies, University of Ioannina, Ioannina 45110, Greece; karagdimitra@hotmail.com (D.K.); pkatapo@gmail.com (P.K.)

**Keywords:** oleaginous microorganisms, lipid accumulation, fatty acid profile, biodiesel production, microalgae, nutraceuticals, omega-3 fatty acid

## Abstract

Microorganisms are known to be natural oil producers in their cellular compartments. Microorganisms that accumulate more than 20% *w/w* of lipids on a cell dry weight basis are considered as oleaginous microorganisms. These are capable of synthesizing vast majority of fatty acids from short hydrocarbonated chain (C6) to long hydrocarbonated chain (C36), which may be saturated (SFA), monounsaturated (MUFA), or polyunsaturated fatty acids (PUFA), depending on the presence and number of double bonds in hydrocarbonated chains. Depending on the fatty acid profile, the oils obtained from oleaginous microorganisms are utilized as feedstock for either biodiesel production or as nutraceuticals. Mainly microalgae, bacteria, and yeasts are involved in the production of biodiesel, whereas thraustochytrids, fungi, and some of the microalgae are well known to be producers of very long-chain PUFA (omega-3 fatty acids). In this review article, the type of oleaginous microorganisms and their expertise in the field of biodiesel or omega-3 fatty acids, advances in metabolic engineering tools for enhanced lipid accumulation, upstream and downstream processing of lipids, including purification of biodiesel and concentration of omega-3 fatty acids are reviewed.

## 1. Introduction

The world’s energy threats have already appeared as a consequence of rapid population growth, extremely unbalanced supplies of food, diminished stockpiles of petroleum resources, and reduced natural resources [1]. Maintaining the sustainable and economic growth of a country with the use of existing and renewable energy sources is crucial when it comes to reducing oil imports [2]. Biodiesel is the most sustainable and renewable substitute for fossil diesel fuel among biofuels that are focused on biomass [3]. It is usually formed chemically by transesterification, where triacylglycerides (TAG), regardless of their origin, interact with short-chain alcohols (generally ethanol/methanol) to form alkyl esters (methyl/ethyl esters) [4]. Biodiesel could be used for the same standard diesel engines irrespective of their origin as well as the feedstocks (it can be microbial oils, vegetable oils or animal fats) through which it arises. The key features that make it environmentally sustainable and eco-friendly are reduced CO_2_ emission levels without sulfur and aromatic content [5]. The use of biodiesel is a sustainable approach to protect the atmosphere from CO_2_ emissions, as well as to play an important role in global climate issues, as it leads to the reduction of greenhouse gases [6]. The use of oils derived from edible resources for large-scale biodiesel production is not a sustainable practice owing to high demands in the food sector [7]. A search for novel non-edible resources is necessary to meet this requirement. The oils from non-edible crops, waste cooking oils from food industries, and animal tallow are the other feedstock for biodiesel production that can reduce the feedstock costs [7]. However, the oils derived from waste cooking oil or other waste sources need some refinement before consideration as feedstock for biofuel production [8,9,10,11]. Waste cooking oil is mainly composed of lipids, including triacylglycerides (TAG), and to a lesser extent of diacylglycerides (DAG) and monoacylglycerides (MAG). It also contains a high amount of free fatty acids (FFA), which severely affect the age of oils in terms of oxidative stability [12]. Nowadays, microbial oils, also known as single-cell oils, are considered as the most efficient feedstock for biofuel production due to their similarities with vegetable oils [13]. Microbial oils have several advantages such as productivity is usually higher than the plants or vegetable oils, easier upstream and downstream processing, easy genetic modifications for the specific products, and they can easily grow in a controlled environment without being dependent on the climate [14,15].

**Figure 1:**
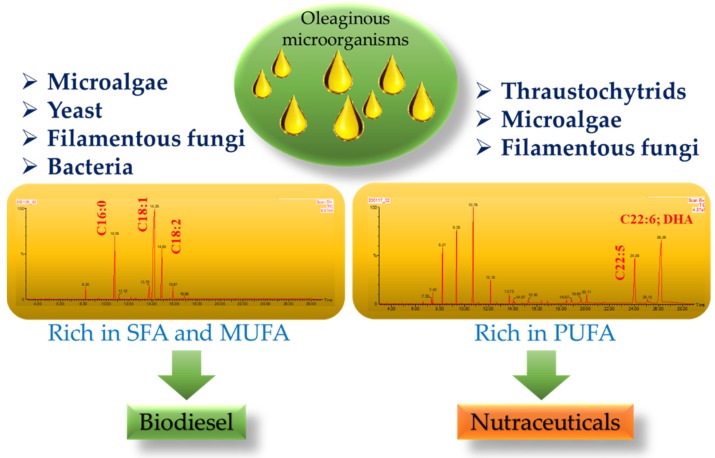
On the basis of the fatty acid profiles, oleaginous microorganisms can be used for biodiesel production or nutraceuticals. Some oleaginous microorganisms such as microalgae, yeast, fungi, and bacteria are rich in saturated and monounsaturated fatty acids in their oils, while some of them are a good source of polyunsaturated fatty acids such as thraustochytrids and microalgae.

Several species of microalgae, bacteria, fungi, and yeast can synthesize more than 20% *w/w* of lipids on the basis of cell dry weight in their cellular compartment and are known as oleaginous microorganisms [13]. Certain species can synthesize lipids up to 70% *w/w* on a cell dry weight basis depending on the cultivation conditions, such as under high C/N ratio [16]. The majority of lipids synthesized by oleaginous microorganisms are of 4 to 28 unbranched carbon chain length [17]. It can be saturated or unsaturated fatty acids depending on the nature of the hydrocarbonated chain, while it can be monounsaturated or polyunsaturated fatty acids (MUFA and PUFA) depending on the number of double bonds [18]. On the basis of the fatty acid profiles of oleaginous microorganisms, they can be utilized either for biodiesel production or for nutraceuticals (Figure 1). A very interesting investigation was carried out by Tchakouteu et al. (2014) to show the interaction between intracellular total sugars (ITS) and lipid synthesis in oleaginous yeast *Cryptococcus curvatus* [19]. When this strain was cultivated in lactose and sucrose under nitrogen-limited conditions, it accumulates high quantity intracellular total sugars up to 68% *w/w* at the initial stage of fermentation, while ITS dropped to 20% at the end of fermentation [19]. In nitrogen-excess conditions, ITS were produced in significant quantities despite the continuous presence of nitrogen in the medium [19]. This interaction between the synthesis of intracellular total carbohydrates and cellular lipids was also reported in the oleaginous microalga *Chlorella* sp. strain growing autotrophically under constant illumination conditions in an open-pond-simulating photobioreactor [20].

After accessing the fatty acid profiles, the lipid obtained from various oleaginous microorganisms was deemed not suitable for biodiesel feedstock due to the high proportion of PUFA in their total lipid content [21]. These PUFA with more than two double bonds are readily susceptible to autooxidation that makes biodiesel technically unsatisfactory, with unpleasant odor [22,23]. Many of the fatty acids can be synthesized in human bodies; however, there is a group of PUFA such as docosahexaenoic acid (DHA) and as eicosapentaenoic acid (EPA) that cannot be synthesized by human beings due to lack of some desaturases and elongases that take part in the synthesis of EPA and DHA from parent omega-3 fatty acids such as α-linolenic acid (C_18:3_ n−3, ALA) [24]. Although these fatty acids can be converted from parent omega-3 fatty acids, their conversion rate is too low to fulfill the daily intake requirement, hence they must be taken from outside diet sources [25]. DHA and EPA have various important roles in metabolic and immune activities and a crucial role in health benefits related to neuro and cardiovascular diseases [26,27]. It also has several advantages in diabetes mellitus and inhibiting tumor cells [28]. It has been already studied that the dietary intake of fish or fish oil can reduce the risk of several types of cancer such as colorectal and prostatic cancers [29]. Nutrition specialists and dieticians suggested that the required ratio of n-6 and n-3 PUFA should be 5:1 or less. The majority of PUFA providing fish in the human diet is species from Salmonidae, Scombridae, and Clupeidae families that have high EPA and DHA content [25]. Fish oil has a high amount of PUFA compared to seed oils and microalgae [30]. However, overfishing has become a persistent problem for the global aquatic ecosystem and killing fish for PUFA is not a sustainable option to fulfill the ever-rising global demand for omega-3 fatty acids [31]. The PUFA obtain from non-vegetarian sources are not consumable for the increasing vegetarian population. To eliminate the issues related with fish oils, the exploration of a sustainable resource of PUFA has been gaining interest in recent years. Vegetable oils can also be considered as an alternative source of only linoleic (C_18:2_ n−6, LA), ALA and arachidonic acid (C_20:4_ n-6, AA) but the large chain PUFA such as DHA and EPA are unable to be synthesize by plants due to lack of elongases and desaturases [32]. Some engineered plants such as *Brassica juncea, Arabidopsis thaliana,* and *Camelina sativa* are good sources for LC-PUFA; however, utilization of these transgenic crops is under consideration of regulatory authorities and social rivalry [33]. Microorganisms are considered as a natural source of omega-3 PUFAs [34]. Oleaginous microorganisms, except filamentous fungi, grow as single-cells, a morphology that is suitable for easy handling in large-scale cultivation conditions [35]. However, despite the promising outlooks of microbial lipids productions for both biodiesel and nutraceuticals, the production cost often hinders their industrial implementation. In a techno-economic analysis concerning biodiesel production from microbial lipids, it was found that the cost of the glucose used as a carbon source can account for 80% of the total material cost [36]. To reduce the cost of the carbon source, an alternative to glucose, low-cost carbon sources have been extensively studied in the literature. Among them, the use of residual lignocellulosic biomass such as agricultural and forest residues, as well as energy crops, is extensively studied in the literature [37,38,39,40,41,42,43,44,45]. Another well-studied alternative carbon source is glycerol. Glycerol is the main by-product of biodiesel manufacturing and has also been extensively studied in the literature as a carbon source for the cultivation of oleaginous microorganisms [46,47,48,49]. Despite the cost of the carbon source, there are already examples of pilot-scale industrial production of microbial oils such as the plant of Neste Oil in Porvoo, Finland [37]. Considerations concerning scaling-up of microbial lipids production in pilot and semi-pilot scales have been extensively discussed in the review article of Athenaki et al. [37].

**Figure 2:**
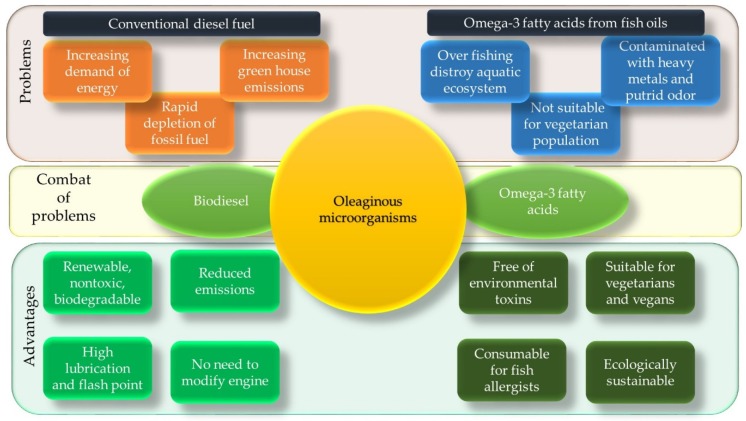
Role of oleaginous microorganisms to combat the problems of greenhouse gas emissions and improving air quality by using biodiesel in vehicles; likewise production of polyunsaturated fatty acids to fulfill the ever-rising global demand of omega-3 fatty acids and replace the use of fish oil that have become a persistent problem for the global aquatic ecosystem.

In this article, oleaginous microorganisms involved in the production of biofuels and nutraceuticals are discussed. An illustrative diagram is presented to show the advantage of using biodiesel and omega-3 fatty acids from microbial oil to combat the problem associated with the utilization of conventional diesel fuel and omega-3 fatty acids from fish oils (Figure 2).

## 2. Oleaginous Microorganisms Used for Biofuel Production

There are three major groups of microorganisms, namely, microalgae, yeast, and filamentous fungi, and finally, bacteria, that can accumulate high content of lipids (>20% *w/w* on the cell dry weight basis) in their cellular compartments and are considered as oleaginous feedstock for biofuel production [50]. A list of oleaginous microorganisms cultivated on various substrates for biodiesel production is presented with their lipid content in Table 1.

### 2.1. Oleaginous Microalgae

Oleaginous microalgae are a promising source for the production of renewable biofuels owing to their efficient photosynthesis capabilities, the reduced needs for growth area compared to terrestrial plants, and their ability to channel the majority of their energy into cell division, which enhances biomass productivity [51]. Microalgae can use both inorganic and organic carbon sources through four different modes of cultivation, namely, autotrophic, mixotrophic, heterotrophic, and photoheterotrophic [52,53,54]. Synthesis of TAGs in microalgae takes place mainly in the sub-cellular compartments such as chloroplast and endoplasmic reticulum as a result of multiple enzymatic reactions [55]. Fatty acid synthesis in the chloroplast, assembly of glycerolipids in endoplasmic reticulum, and accumulation of TAGs into the oil bodies are the three major steps involved in the accumulation of lipids in microalgae [56]. It has been proven that different stress conditions such as physical, chemical, or environmental, individually or in combination, facilitate the synthesis of high amounts of lipids [57]. Under different stress conditions, microalgae can switch their metabolism towards the formation and accumulation of neutral lipids in the form of TAGs, which serves as a form of carbon and energy storage [58,59,60,61,62]. Microalgae synthesize lipids via the de novo pathway, which starts in the chloroplast by CO_2_ fixation into sugars, which are further metabolized to form acetyl-CoA, which is a precursor of fatty acid synthesis [57,63,64]. Photosynthetic reactions occurring in autotrophic cultivation provide not only a carbon source but also assist in generating reducing power (NADH and NADPH) that is finally used for lipid synthesis [65]. However, low biomass and lipid productivity and the requirement of appropriate photobioreactors are major drawbacks of industrial-scale applications of autotrophic cultivation [55,56,57,58,59,60,61,62,63,64,65,66,67]. In the past decades, researchers have been focusing more on the heterotrophic cultivation of algae as it has many advantages over the photoautotrophic cultivation, such as cost-effectiveness and being relatively easy to cultivate with quite low daily maintenance [54,55,68]. Furthermore, heterotrophic cultivation can be carried out in any fermenter that is utilized for yeast and bacteria without illumination, and as such, the use of photobioreactor is not required, which in turn reduces the overall production cost [68]. Glucose is a commonly used carbon source for the heterotrophic mode of cultivation; however, it must be obtained from renewable sources to avoid the high cost associated with feedstocks [66]. Various inexpensive raw materials obtained from inedible lignocellulosic biomass from forests such birch, spruce, and beech, or agricultural residues such as rice and wheat straw, sugarcane bagasse, corn stover, waste molasses, soy whey, and industrial wastewater, have been successfully applied to support heterotrophic cultivation [69,70,71,72].

### 2.2. Oleaginous Yeast and Filamentous Fungi

Typically, oleaginous yeasts are chosen when it comes to the production of lipids. Oleaginous yeasts are well-studied microorganisms and include species of the genera *Candida, Rhodosporidium, Yarrowia, Cryptococcus*, *Rhodotorula*, *Lipomyces,* and *Trichosporon*, some of which can accumulate lipids up to 80% *w/w* of their dry cell weight [73,74]. Additionally, the lipid metabolism of these oleaginous yeasts is well-known [14,16]. Other potential strains for lipid production are continuously sought for and selected, with several strains engineered for increased lipid production [75,76,77,78]. There are certain criteria that these strains should meet, such as the ability to grow to high cell densities along with high lipid content on various carbon sources and robust process conditions [79,80,81,82]. To improve economic feasibility, oleaginous yeast strains have thus been cultivated on various non-food competing carbon sources, such as lignocellulosic materials [42,44,74,83,84,85,86]. The non-oleaginous yeast *Saccharomyces cerevisiae* is used in many industrial applications since it is easy to cultivate and its genetic tools are well-established. Consequently, *S. cerevisiae* has also been exploited and subjected to metabolic engineering approaches for lipid production [87,88,89,90,91,92,93,94].

Oleaginous filamentous fungi are promising microbes for biofuel production and have certain advantages such as unique fatty acid profiles with fatty acids such as γ-linolenic acid (GLA) that cannot be synthesized in high amounts by other oleaginous microorganisms [95,96]. Fungi can be cultivated on inexpensive feedstocks such as waste molasses, monosodium glutamate wastewater, sewage sludge, glycerol, and agricultural residues [97,98,99,100,101]. An oleaginous fungus, *Cunninghamella echinulate*, cultivated on orange peel and glucose, synthesized 46.6% of total lipids, including 14.1% of γ-linolenic acid (GLA) [102]. Similarly, the fungus *Mortierella alpina* LPM 301, cultivated on glucose with potassium nitrate, synthesized high amounts of lipids (31.1%) that contained 60.4% of arachidonic acid (ARA) [103]. Oleaginous fungi were used to grow along with microalgae for enhanced lipid productivity, e.g., a marine microalgae *Nannochloropsis oceanica* and an oleaginous fungus *Mortierella elongata* were co-cultivated to initiate bio-flocculation that yielded high amounts of TAGs and PUFAs (polyunsaturated fatty acids), along with total lipids [104]. *Aspergillus niger* cultivated on sugarcane distillery wastewater or vinasse as low-cost feedstock was utilized for the production of biodiesel [105]. In a study, *A. niger* cultivated on pure vinasse showed highest cell dry weight of 24.05 g/L, while the highest lipid produced (2.27 g/L) was by *Aspergillus awamori* among 28 different strains tested [106].

### 2.3. Oleaginous Bacteria

Oleaginous bacteria are also a good source of TAGs; however, their utilization for biodiesel production is limited compared to microalgae and yeast [50]. Some important genera of oleaginous bacteria are *Rhodococcus* sp., *Gordonia* sp., *Acinetobacter* sp., and *Arthrobacter* species. Among them, *Rhodococcus* sp. has been the most extensively studied as a result of their ability to grow on versatile substrates [107,108]. Within the biorefinery concept for the production of biofuels, lignin is often left underutilized. Only certain fungi (mainly white-rot fungi) and prokaryotes have lignin-depolymerizing enzymes [109]. Recently, *Rhodococcus* sp. was studied for its potential to degrade lignin and finally assimilate lignin monomeric compounds into the lipid accumulation pathway [110,111]. In a study, *Rhodococcus opacus* attained a lipid content of 26.8% *w/w* when cultivated on aromatics obtained from organosolv pretreatment of loblolly pine along with lignocellulosic pretreatment effluents containing various sugars [112]. This species was also applied to convert oxygen-pretreated Kraft lignin into valuable lipids [107].

## 3. Oleaginous Microorganisms Used for Nutraceuticals Production

Oleaginous microorganisms such as thraustochytrids, microalgae, and filamentous fungi rich in PUFA were considered for nutraceutical production. A list of oleaginous microorganisms and their EPA and DHA content are presented in Table 2.

### 3.1. Oleaginous Thraustochytrids

Thraustochytrids are a heterotrophic fungus-like clade of Stramenopiles but are often inadequately referred to as “algae”. Algae is a term used to refer to photosynthetic organisms, often excluding Embryophyta and Prokaryotes, for instance, Cyanobacteria [132]. The misleading term of being “algae” is possibly due to higher marketing value, as “green, healthy and sustainable” is more likely associated with algae rather than fungi or yeast, for example.

Thraustochytrids are a good source for commercial production of DHA. Due to an increased demand for DHA, researchers are developing new and improved technologies for the production of DHA by thraustochytrids. Generally, higher temperatures (25–30 °C) favor optimal growth, while lower temperatures stimulate the DHA production at the expense of reduced growth. This, in turn, results in an overall low yield of DHA [133]. To overcome this, different cultivation techniques can be applied, such as growing the thraustochytrids initially at higher temperatures (approximately 25 °C) to stimulate their growth and then switch to lower the temperature (15 °C) to enhance the DHA production [134]. It has been documented that the species have a wide pH tolerance, ranging between 5 to 8 for the growth and production of DHA. Furthermore, the salinity optima and tolerance levels of thraustochytrids are different between the strains; some are even able to grow at low salinities of 2 ppt [135]. These characteristics make thraustochytrids advantageous as the strain is tolerable to several different cultivation conditions. *Schizochytrium* spp. are a well-studied thraustochytrid with the ability to produce approximately 35%–40% *w/w* of their total fatty acid as DHA. *Schizochytrium* spp. are currently used commercially for their production of PUFA [136]. Recently, studies have been conducted to investigate the potential of growing marine species *Aurantiochytrium sp.* T66 (ATCC PRA-276) in heterotrophic cultivation using forest biomass hydrolysates (30 g/L glucose) in flasks. The study obtained results of cell dry weight and total lipids of 10.39 g/L and 4.98 g/L, respectively, of which 25.98% constituted of DHA [23]. In the same work, *Aurantiochytrium sp.* T66 was also cultivated in a bioreactor and resulted in elevated values for the cell dry weight (11.24 g/L), total lipid (5.90 g/L), and DHA content (35.76% of the total lipids) [23], enabling a better process control. These results indicate the great potential in valorizing sustainable resources for the production of DHA.

### 3.2. Oleaginous Microalgae and Diatoms

Marine microorganisms are well developed to be metabolically efficient due to their diverse environmental adaptations. They are therefore capable of producing unique microbial metabolites, and they have also evolved to use limited dissolved organic matter in a ratio where more metabolites are produced than energy being consumed [136]. Biosynthesis of fatty acids is species-specific and is dependent on different cultivation techniques [137,138]. It is possible to enhance the lipid content and the production of certain fatty acids in culture by applying different specific abiotic factors, although conditions that yield high lipid content might not be the most favorable for growth [138]. The best well-known strategy to achieve high lipid accumulation in oleaginous microorganisms is through nitrogen starvation [139,140,141,142,143,144,145]. Nitrogen is an essential part of the synthesis of proteins, nucleic acids, and chlorophyll, and during nitrogen limitation, microalgae undergo rapid metabolic remodeling, directing the carbon flow toward lipid production [140,144,146,147,148,149,150,151,152,153].

Different marine microorganisms have been exploited for their potentials to produce nutraceutical value fatty acids [136,154,155]. Marine microalgae have a higher content of PUFA compared to freshwater species since the marine species need to produce more unsaturated fatty acids to survive in the marine environment [136]. Thus, it might be of higher economic interest to cultivate marine microalgae. For example, the marine oleaginous diatom *Fistulifera solaris* cultivated in photoautotrophic conditions has been reported to produce high amounts of EPA. A study was carried out with the aim of *F. solaris* photoautotrophically producing EPA at the same level as heterotrophic fungi cultures. With optimized EPA production conditions, *F. solaris* has been reported to yield a production of 135.7 mg/(L·day), which is the same order of magnitude as can be obtained from heterotrophic cultivation [156]. In comparison to this, it has been reported that the heterotrophic growth of the marine diatom *Nitzschia laevis*, supplemented with glucose, resulted in EPA production of 174.6 g/(L·day) [157].

The diatom *Phaeodactylum tricornutum* is photoautotrophic in nature, while it can be grown in mixotrophic conditions, allowing photoautotrophic as well as heterotrophic cultivation conditions simultaneously, although, in this case, glucose utilization requires light [158,159,160,161,162,163,164,165,166,167]. When glucose transporters were introduced through genetic engineering, *P. tricornutum* could grow heterotrophically on various carbon sources in the dark [166,168]. *P. tricornutum* showed enhanced values of EPA and DHA productivities when grown mixotrophic, as compared to the obtained results from photoautotrophic growth. It has also been shown that using birch and spruce hydrolysates as a glucose source, *P. tricornutum* generated 3.11- and 3.2-times higher EPA productivity, respectively, compared to photoautotrophic cultivation [21]. Another significant candidate is the heterotrophic marine microalgal species *Crypthecodinium cohnii,* which has been used for commercial production of DHA [168,169,170,171,172,173,174]. *C. cohnii* is unique due to DHA being almost the only PUFA present in its lipid profile, and DHA content can be up to 65% of the total fatty acids [175]. This characteristic makes the purification process of DHA from *C. cohnii* very attractive.

In the late 1980s, a group at Martek Biosciences produced DHA-rich oil from *C. cohnii* for the infant formula industry, with the requirement that it should have a high content of DHA and be free from EPA [176]. An advantage that is common for both *C. cohnii* and the earlier-mentioned thraustochytrids is that they are heterotrophic organisms and can be grown to yield high biomass in fermenters [132]. A major limitation of photoautotrophic cultivation is the effect of self-shading. For example, pond-grown algae generally achieve a culture density of approximately 0.5 g/L, after which they become self-shading and, subsequently, the biomass production becomes limited [177]. Hence, the low productivity prevents their commercialization [22]. Depending on the latitudes of interest, it is necessary to consider species that can be cultivated at the location to avoid expensive heating and artificial lighting [178]. The diatom strain *Leptocylindrus danicus* grows at temperatures at 8 °C, for example, while it also has shown a constant biomass content for a wide range of temperatures, possibly qualifying to be cultivated throughout the year [137]. Even though the phototrophic cultivations fix CO_2_ and emit O_2_, heterotrophic and mixotrophic cultivation conditions are considered more advantageous regarding the high productivity of lipids that can be achieved as compared to the photoautotrophic yield. However, this is an economic disadvantage due to the high costs of organic carbon sources. The use of other sources of sugars has been suggested to reduce these production costs, for example, the use of industrial waste and non-edible lignocellulosic materials [179].

### 3.3. Oleaginous Filamentous Fungi and Yeast

The first microbial strain known for producing commercial γ-linolenic acid-rich oil was *Mucor circinelloides*, a filamentous fungus [180]. The oleaginous fungus *Morteriella alpina* 1S-4 is a good source for AA production; it is able to produce EPA and AA through the n-3 and n-6 PUFA biosynthetic pathways, respectively. *M. alpina* can produce high amounts of ARA even when grown with very low concentrations of glucose (2%). The strain has been used to commercially produce ARA-rich oil for infant formula applications since the late 1980s [176]. Further examples of marine filamentous fungal species are *Penicillin sp., Epicoccum sp.,* and *Keissleriella sp.*, which have been reported to be promising producers of bioactive exocellular polysaccharides (EPS) [135]. Cultivation of genetically engineered oleaginous yeast strain *Yarrowia lipolytica* has been reported to yield EPA productivity of 161.04 mg/(L·day). *Y. lipolytica* is a well-studied strain due to its biotechnological characteristics such as accessibility for genetic manipulation, as well as a unique ability to grow on hydrophobic substrates [181,182]. *Y. lipolytica* is labelled as “generally recognized as safe” (GRAS) and is, therefore, an attractive host for the manufacture of nutraceuticals [176]. The cultivation of yeasts has the advantage of offering enhanced productivity with the use of cheap substrates such as waste glycerol or sugars derived from lignocellulosic biomass, making it economically favorable [183,184].

## 4. Ex-Novo Lipid Synthesis When Oleaginous Microorganisms Are Cultivated on Hydrophobic Substrates

Some oleaginous yeasts have the unique ability to synthesize a different fatty acid profile than those presented in the hydrophobic medium containing saturated and unsaturated fatty acids. Oleaginous yeasts utilize hydrophilic substrates as a preferable carbon source for lipid accumulation via the de-novo pathway, while a few of them are reported to survive in the hydrophobic environment and show lipid synthesis via the ex-novo lipid synthesis pathway. *Yarrowia lipolytica* is considered as a model oleaginous microorganism to understand the mechanisms behind the uptake of hydrophobic substrates [205]. Some other oleaginous yeasts such as *Cryptococcus*, *Rhodosporidium*, *Geotrichum,* and *Trichosporon* have also been explored to cultivate on hydrophobic substrates [7,82]. They can assimilate free fatty acids, TAGs, and alkanes with the help of several multigene families that contribute to the catabolic and metabolic route to degrade a wide range of hydrophobic substrates [35]. Extracellular lipases secreted by the oleaginous yeast help to improve the assimilation of the hydrophobic substrates by degrading it into free fatty acids and assimilated into the yeast cells by active transporters or by simple diffusion depending on the concentration gradient [206,207]. The hydrophobic substrates are utilized for the growth of microorganisms or for storage in the form of lipid droplets, where the lipid composition can be similar to substrate, or they can change the lipid composition [208,209]. The hydrophobic materials utilized as feedstock for biomass and lipid production by oleaginous yeast can be free fatty acids obtained from an industrial waste stream, waste cooking oils, effluents from dairy- and butter-producing industries and waste fish oils [206,207,208,209,210,211]. All these hydrophobic substrates are internalized in the cellular compartment of oleaginous yeast by creating certain changes on the surface, such as protrusions that help to increase the contact area between the hydrophobic substrate and the yeast [209,212].

## 5. An insight into Role of Microbial Lipids as Exotic Fats and Cocoa-Butter Substitutes

Cocoa butter (CB) is a value-added product of cocoa bean processing industries that is obtained from *Theobroma cacao* plant. The major constituents of cocoa butter are high saturated fatty acids with less amount of highly unsaturated fatty acids, and the composition is totally dependent on plant variety and the culture conditions [213]. The common fatty acids profile of cocoa butter is C16:0 23%–30%; C18:1 30%–37%; C18:0 32%–37%; C18:2 2%–4%. Due to the increasing demand and supply shortage, there is an increasing interest in cocoa butter alternatives. Various approaches have been made to produce similar lipid composition with cocoa butter, such as using a mixture of different fats from exotic plants (illipe’ butter, mango fat, kokum butter, sal fat) and palm oil; however, this strategy fails due to high cost of exotic fats itself [214,215,216]. Producing cocoa butter substitutes by a biotechnological approach such as enzymatic and fermentative production has already been discussed [215,217,218].

Yeast lipids are considered as one of the major microbial substitutes of cocoa butter for industrial applications [219,220]. Under this approach, the stored lipids in the form of TAGs are esterified in the sn-2 position by unsaturated fatty acids [219,221]. Oleaginous yeast synthesizes lipids enriched with unsaturated fatty acids, while the composition of cocoa butter composition contains 60% (*w/w*) of saturated fats (palmitic and stearic acid) [222]. To increase the content of these fatty acids, several approaches have been tried, such as genetic manipulation, growth in high stearic acid rich medium, use of Δ9 and Δ12 desaturase inhibitors [223], and low oxygenation of the growth medium [224]. *Y. lipolytica* can incorporate C12:0, C12:0, C14:0, and C16:0 easily while it is hard to ingest C18:0 as a substrate, and, moreover, the uptake of substrate ceased when C18:0 was present in abundant [225].

Similar results were also observed with *Y. lipolytica* cultivated on a mixture of saturated and unsaturated fatty acids [213,225,226], and the same results were obtained from bacteria *Pseudomonas oleovorans* cultivated on fatty acids and 3-hydroxyalkanoic acids [227].

## 6. Application of Metabolic Engineering Technologies to Improve Lipid Production by Oleaginous Microorganisms

Microbial sources for lipid production that can be used for energy or nutraceutical purposes are gaining significant attention. Oleaginous microalgae, fungi, and yeasts constitute perfect candidates as they fulfill the product demands in parallel, avoiding the controversy of using edible sources. The understanding of biochemical and metabolic mechanisms related to biosynthesis and accumulation of fatty acids is the first step in order to enhance their production. Placing emphasis on differences among the microorganisms and aiming for the most suitable metabolic spot leads to improvements in lipid yields and modification of lipid profiles. The wild-type strains can be modified to improve the lipid accumulation by using the recent metabolic engineering tools [77,161,228,229,230,231,232,233,234,235,236,237]. The methods of metabolic engineering technology interfere at parts or whole of the proteome, lipidome, transcriptome, genome, and metabolome of microorganisms. Therefore, the understanding of these paths is necessary for the control of lipid production and the design of modified strains [238]. A first step towards this direction is the use of modeling tools that could predict the difference in the behavior of every change and could help the design of the most suitable modification. Except for the enhancement of lipid production, time and cost are basic parameters of an effective modification. Systems biology, in combination with synthetic biology and evolutionary engineering, provide these tools [239]. In addition, constrain-based models, together with genome-scale metabolic models, provide a relationship between genotype and phenotype and, as a result, novel genetic designs, prediction of signaling network processes, and prospective experimentation [240,241,242].

Every oleaginous microorganism has different lipid production capabilities, and there are many ways to alter and enhance the lipid metabolism and lipid production. Scientists can manipulate the pathways related to the synthesis, storage, and profile of lipids. They can also modify the pathways related to the adaptivity of microorganisms to the environment that result in changes in product production rates and amounts. In the next paragraphs, some of these changes will be reported. The major directions that enclose subsections can be classified into the overexpression of genes or enzymes of biosynthesis pathways, suppression, blocking or knockout of genes of competitive pathways, regulation of bypass pathways, multi-gene approaches.

**Figure 3:**
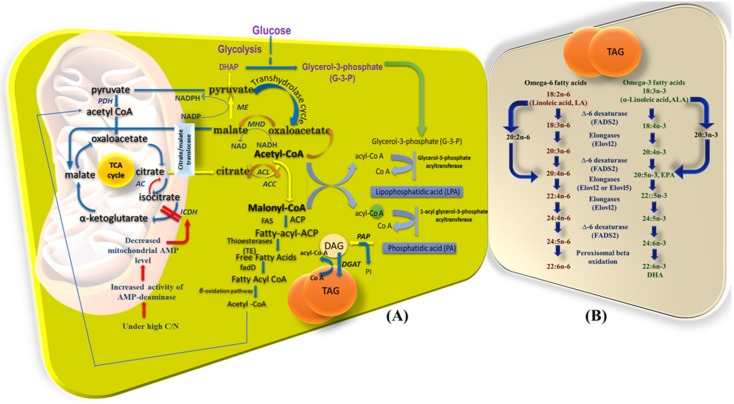
(**A**) *De-novo* fatty acid synthesis in oleaginous microorganisms (adapted from [13,16,18,221,243,244]), and enzymes involved in lipid accumulation. AC, aconitase; ACC, acetyl-CoA carboxylase; ACL, ATP-citrate lyase; ACP, acyl carrier protein; FAS, fatty acid synthetase; ICDH, iso-citrate dehydrogenase; MD, malate dehydrogenase (cytoplasmic); PD, pyruvate dehydrogenase; PAP, phosphatidic acid phosphohydrolase; DGAT; diacylglycerol acyltransferase; FAS: fatty acid synthase. (**B**) Biosynthesis pathway of omega-3 and -6 fatty acids (EPA and DHA) from parent fatty acids (LA and ALA) through a series of desaturation and elongation reactions [17,161,245,246].

A basic synthetic pathway for fatty acids in oleaginous microorganisms is presented in Figure 3. Summarizing the fatty acid metabolisms in bacteria, acetyl-CoA constitutes the central molecule. It leads to the formation of malonyl-CoA, followed by the production of fatty acyl carrier proteins (fatty acyl ACPs or fatty acyl moiety), and finally transformation into free fatty acids with the help of thioesterases [247]. ACC (acetyl-CoA carboxylase) catalyzes the first step, while FAS (fatty acid synthases) plays a major role in the biosynthetic pathway [248]. Similarly, in microalgal cells, acetyl-CoA is the central molecule, which is catalyzed to malonyl-CoA, which is further catalyzed to malonyl-ACP with the help of ACP. This molecule is transformed to free fatty acids with the contribution of KAS (ketoacyl-ACP-synthase) and FATA (acyl-ACP thioesterase). Free fatty acids can be evolved to PUFAs with the help of specific desaturases and elongases [249]. In yeasts, acetyl-CoA is transported to cytosol, where it is catalyzed to malonyl-CoA with the help of ACCs, which concludes to FAs [250]. For all the microorganisms, we should mention that TAG synthesis follows the Kennedy pathway, which takes place in the endoplasmic reticulum or lipid body membranes. In this pathway, acetyl-CoA is transformed into TAGs through a number of phosphorylation and dephosphorylations, where enzymes like acyltransferases (DGAs), ketoglutarates (KGs), and dehydrogenases play important roles [251].

One of the metabolic engineering methods is the successful expression or overexpression of key enzymes. Based on the lipid biosynthesis pathways, overexpression of genes that encode ACC and FAS is among the first choices. In some cases, the co-expression of more genes is necessary for the successful increase of lipid synthesis, as later steps of the pathway could limit the previous results. For instance, acyl-ACP could inhibit the overexpression of ACC in *E. coli* cells [248]. Similarly, in the case of TAG synthesis improvement, overexpression of genes for key enzymes in the Kennedy pathway, like DGA and KGs, constitutes a proper choice. Another technique is the regulation of bypass pathways. In this method, scientists intervene in genes that regulate molecules that do not exist in basic lipid biosynthetic pathways. For example, in *E. coli* cell overexpression of ACS genes could result in an increase of acetate formation, which results in enhanced activation of acetyl-CoA and, as a result, lipid synthesis [252]. On the other hand, the suppression or knock out of genes that are related to lipid oxidation, degradation, and their synthesis inhibition is another commonly used approach. In this context, inactivation or dysfunction of enzymes that are responsible for β-oxidation, such as acyl-coenzyme oxidases (AOX) via knock-out of their genes (*POX*) [253], leads to improved accumulation of lipids. In the case of TAC synthesis improvement, scientists could block the phospholipid biosynthetic pathway [254]. As the research on gene metabolic engineering is evolving, the combination of more than one of the above methods is taking place. The multi-gene approach, in which more than one gene of key points of lipid metabolism is overexpressed, or some are overexpressed in combination with knock-out of others, is proposed [250,255]. For example, by introducing four modifications in the *E. coli* genome, Lu X et al. increased the lipid production by about 20 times. More specifically, they overexpressed three genes and knocked-out one [247]. By knocking out the acyl-CoA synthetase, they stopped the degradation of fatty acids, while overexpressing ACC produced more malonyl-CoA. Finally, by overexpressing two thioesterases (an endogenous and an exogenous), they increased the short chain FAs and decreased the inhibition from fatty acyl-ACPs. As such, Lu’s team increased the production of lipids suitable for biodiesel [247]. A similar study in microalgal cells and specifically on *Haematocccus* showed that the application of metabolic engineering techniques to more than one key gene, in combination with changes in environmental conditions, could result in better quality and quantity of value-added products. Expression of key genes related to ACP, KAS, and FATA could affect both monounsaturated and polyunsaturated fatty acid synthesis [249]. A similar study has been achieved in the oleaginous yeast *Y. lipolytica*, with the overexpression of the two key genes *ACC1* and *DGAT1*. When these genes were overexpressed separately, they led to a 2-times and 4-times increase of lipid production, respectively. When their overexpression was combined, they resulted in a 5-times greater lipid accumulation compared to control, indicating their synergistic effect [250]. As was shown, the multi-gene approach already delivers great results among all the categories of microorganisms, leading to enhanced fatty acid production for their use in nutritional or energetic purposes. For the above-mentioned gene modifications, where the suppression or activation of specific genes is required, there exist bioengineering methods like mutagenesis, homologous recombination, the use of micro RNA (miRNA), and short interfering RNA (siRNA) [256]. All these modifications and the selection of the most appropriate tools are strongly dependent on the type of microorganism, the strain, their genetic profile, and the desired result.

So, it is obvious that the first step is the understanding of metabolic pathways that take part in lipid metabolism. After that, scientists have in their hands a plethora of tools for the prediction, design, test, and creation of advanced oleaginous microorganisms. It is important to mention that each modification strongly depends on microorganism species and strain. So, the modification should be carefully selected. In all cases, the cost, in combination with the result, should be considered. The only drawback about microorganisms that have passed through metabolic engineering technology is the probability of some impact on the environment and human health in the case of their release and reproduction in natural habitats. Something like this should be checked by specific committees before their widespread use and commercialization [257].

Microbial lipids can be synthesized in two different ways: through the metabolism of hydrophilic substrates, as described above, and through the fermentation of hydrophobic substrates (such as fatty acids, fats, oils, alkanes) [206]. It is important to mention that in ex-novo fermentation, lipids can be accumulated at the same time with growth and is independent of nitrogen and other nutrients supplies [250]. Moreover, in this pathway, lipids can be modified according to the requirements of the microorganisms.

In summary, the hydrophobic substrates are hydrolyzed, and, as a result, they release their oils, creating droplets. These droplets are internalized by cells through transport mechanisms. There, the substrate can be transformed into fatty alcohols through oxidation and then to fatty acids that can undergo the peroxisomal β-oxidation or be stored [258,259]. Some microorganisms, like the yeast *Y. lipolytica*, excrete a compound to the medium called liposan that can make the lipid droplets smaller [260]. Moreover, they are able to produce lipases to hydrolyze external TAGs [16]. Otherwise, substrates are able to transport to the endoplasmic reticulum by direct transport systems, being attached to some protrusions of yeast body [209]. Several genes are involved in the above processes, such as *LIP* genes (lipases/esterases), *ALK* genes (cytochromes P450), and *POX* (peroxisomal acyl-Coa oxidases) [206]. The lipase family contains a great number of lipases, having different substrate preferences from medium to long-chain FAs [207,261]. *ALK* genes also constitute a big family, containing different ALK for different chain length alkanes [207,261]. Depending on the length of the chain of the acyl-CoA, there are also different acyl-CoA oxidases for the yeast *Y. lipolytica*, and different POXs.

A most usable strategy in order to increase the lipid accumulation through ex-novo cultivation is reducing lipid catabolism. It can be achieved by knocking out genes related to β-oxidation [34,212]. The most common among them are the *POX* genes that encode acyl-CoA oxidases (AOXs). The limitation of the oxidation in combination with the deletion of *GUT2* or the over-expression of *GPD1* genes, which are genes related to the glycerol phosphate pool, lead to a lipid content of 80% of dry weight [262]. Knocking out *POX* genes in combination with the engineering of glycerol (through G3P) can lead to a 40%–70% increase in lipid accumulation in the above-mentioned microorganism [250].

The combination of de-novo and ex-novo pathways and the use of metabolic engineering tools could probably lead to even greater accumulation of lipids. Moreover, the advance on metabolic tools related to ex-novo lipid synthesis could lead to the use and recycling of wastes containing oils, reducing their ecological footprint, and producing high-value products.

## 7. Downstream Processing

As mentioned above, the lipids that are produced by microorganisms are referred to as single-cell oil (SCO) and consist of an important feedstock for the omega-3 fatty acid-based and biodiesel-based industries. The downstream process of SCOs production includes the microorganism cultivation, their biomass harvesting (separation of cells from the cultivating medium), the extraction of lipids, and, at the end, their purification. Every step can be achieved through different techniques, each one being preferred depending on the microorganisms and their strain.

### 7.1. Biomass Harvesting; Separation of Cells from the Cultivating Medium

Biomass harvesting consists of a basic step in the downstream process associated with 20%–30% of the total production cost [263]. One of the major challenges is the separation of the usual low rate and small size of cells [263,264]. Moreover, it is important to lead to a slurry that can be used for the extraction of lipids and to water and nutrient recycling, if possible. The harvesting methods can be chemical, mechanical, or biological.

One of the most commonly used methods is centrifugation. Most of the microorganisms can be harvested through this technique, with its efficiency depending on the cell size, the density of the culture, and the time and the speed of the procedure. It is ideal for cells with high-value end products as it favors their recovery [265]. Moreover, it is suitable even for big culture volumes, without the need for extra chemicals. Consequently, it is ideal for cells with high-value end products. The main disadvantage is its high-energy cost and the fact that it may cause damage to sensitive cells because of the high forces [52,265]. Filtration is another technique for biomass harvesting. It consists of an easy method with many limitations. Various filtration methods, such as ultrafiltration, microfiltration, and vibrating membrane filtration, were used to filter the microalgae [266,267,268,269].

The necessary equipment is a filter with the proper dimensions that can retain the cells. As such, conventional filtration is suitable for bigger size microorganisms (>70 μm) with long-length or those that shape big colonies [270,271]. For microorganisms with the dimensions of bacteria, there are special filtrations, such as micro-filtration, membrane-filtration, or ultra-filtration, being suitable only for small volumes [270]. One more limitation of this technique is the cost. Furthermore, as the cells are accumulated, the flux is decreased, leading to membrane fouling/clogging and, in turn, the need to replace the filter [272].

On the other hand, flotation consists of a method for unicellular cells with small dimensions. In this method, the cells are attached to bubbles [273] so the microorganism cells with the bubbles, having a lower density than media, go to the surface where they are collected [274].

Flocculation is also among the commonly used methods of cell harvesting. It is based on grouping the cells because of their surface charges and the developed electrostatic interactions, leading to bigger blogs that are easier to been harvested. The microalgal cells carry a negative charge, and as a result, the cells appear to be electrostatic repulsed and are not allowed to aggregate in the culture [265]. During flocculation, this repulsion is reduced or even disappears with the help of chemical or biological flocculants. The use of aluminum, ferric, and zinc salts towards this aim has been widely reported. These salts are suitable depending on the final product and the cultivated microorganism. In addition to the cost of the procedure, a very important parameter is the cause of culture contaminations and the potential toxic effects on cells and the environment because of the chemical elements used [275]. The evolution of chemical flocculation to avoid the mentioned problems is the use of polymers as flocculants, which can cause physical links among the cells, and the efficiency of the method depends on polymer characteristics such as their molecular weight [265]. In addition, there are natural polymers, like chitosan, that are biodegradable and not toxic, which are capable of harvesting microorganisms of bacterial size [276]. In recent years, bio-flocculation, in which other microorganisms induce the flocculation of the basic one, has gained attention [277]. In this method, the bio-flocculating microorganisms are cultivated together or separately with the basic microorganism. For example, the harvesting of microalgal cells can be achieved with the addition of bacteria of fungal cells [278]. The advantages of bio-flocculation are the reduced cost, the lack of chemical contamination, and the environmentally friendly background. The drawback is the possible microbial contamination [279].

The above-mentioned techniques can be separated into two categories: dewatering thickening (the first two) and dewatering methods. In most of the cases where more than one method was used in combination, this resulted in better cell separation. A nice example is the shaping of microalgal-fungus pellets that are formed with continuous agitation so that the pellets can easily be harvested via filtration [271]. After cell separation, in some cases, drying is the last step of this process. So, the final slurry is “clear” from the media solution.

From all the above, it is obvious that there is no universal method for biomass harvesting. The result is related to the cell properties (morphology, size) of each microorganism and strain, the desired end product and its use, and the used media. In most of the cases, a combination of methods leads to the most efficient and environmentally friendly result. Harvesting is a crucial step as the isolation of the slurry from media enables further downstream processes with the extraction of the desired products [280].

### 7.2. Lipid Extraction Methods

Conventional quantification relies on the solvent extraction of lipids from the cells. Lipid extraction refers to the process of separating neutral lipids from the rest of the cellular matrix and water. To maximize lipid recovery, techniques such as ultrasound, microwave, bead milling, and detergent-assisted extraction have been employed [281,282].

#### 7.2.1. Overview of Extraction Techniques

Sustainable production of biofuels depends largely upon efficient lipid extraction from the microbial cells. The presence of a thick and robust cell wall renders lipid recovery processes complicated, and the high cost and high energy demands involved in the lipid extraction pose a restriction of using microbial biomass as raw material for biodiesel production at an industrial scale [283,284]. To overcome this, there are several pretreatment methods to improve the lipid extraction, making the process more easy, cost-effective, robust, efficient, selective, environmentally friendly, and also considered for large-scale production [285,286], which will be discussed in the next section. The lipids are not a uniform compartment; they can be polar (phospholipids) and nonpolar (triacylglycerol), hence partitioning of lipid classes from the total lipid fraction must be related to differences in their polarity [287,288]. Lipid extraction from microorganisms occurs through two different routes, the dry and the wet route. The wet route of lipid extraction is advantageous over the dry route due to reduced cost and energy demands, which makes the lipid extraction more feasible by eliminating the drying process prior to extraction [57,289]. The Bligh & Dyer and Folch are the two most commonly used methods when it comes to lipid extraction, in which mixtures of chloroform and methanol (2:1 by volume) are used as solvents. However, other issues related to cell disruption for enhanced lipid recovery must be considered [290,291,292].

The Folch method is less time consuming; however, its lower sensitivity compared to the other procedures is the major disadvantage of this technique [293]. The Bligh & Dyer method is more precise to extract lipids, as proteins are precipitated in the interface of two liquid layers that can be further separated from the lipids. The obtainability of pure lipids from the Bligh & Dyer method compared to the Folch method makes the former more suitable to be applied in pilot and large-scale extractions. To improve the lipid recovery, these two conventional lipid extraction methods are modified accordingly by several researchers. Matyash et al. (2008) suggested a modified method of Folch and Bligh & Dyer, where methyl-tert-butyl ether (MTBE) was used as a solvent for the extraction of lipids with better recovery and suitability for the lipidome profile [294]. In other modifications, acidic treatment (HCl) of biomass before applying the Bligh & Dyer method was developed to improve the lipid recovery with more polyunsaturated fatty acids [295]. Although these two methods are commonly used, they involve the use of toxic chemicals (chloroform and methanol), which could pose an environmental threat and a potential threat to human health. As such, switching to less toxic solvents while maintain or improving the extraction efficiency would offer a better option. Toward this direction, 2-ethoxyethanol (2-EE) is very effective for lipid recovery in comparison to the conventional solvents chloroform and methanol or hexane, and is considered environmentally safer [296].

Supercritical fluid extraction (SFE) is another alternative for lipid extraction, offering high extraction ability; however, it has not been used in commercial-scale yet [297]. Recent advances of SFE involve the use of supercritical fluids like ethanol, ethane, ethylene, toluene, benzene, methanol, CO_2,_ and water [298,299]. Among them, CO_2_ is attracting attention for application in the extraction processes of pharmaceutical and health-related products [300].

#### 7.2.2. Pretreatment for Enhancement of Lipid Extraction

As lipids constitute an intracellular compound, a pretreatment step of the microbial biomass is often required as a means to disrupt the cellular integrity of oleaginous microorganisms and improve the lipid extraction efficiency [290]. Apart from enhancing the lipid extraction, the application of pretreatment can also allow lipid extraction directly from wet biomass [301]. Generally, the pretreatment techniques are divided into mechanical and non-mechanical methods, with the non-mechanical methods to be further divided into physical, chemical, and enzymatic methods [285,290,302,303,304]. Currently, various pretreatment methods have been employed in laboratory-scale, such as high-pressure homogenization, bead beating, microwave, ultrasonication, osmotic shocks, and autoclaving. However, none are effective for large scale processes [51], and there is a need to further develop such methods to be cost-effective for industrial applications.

Oil press or expeller press is the simplest mechanical method used for lipid extraction from oily seeds that has now been tested for algal biomass, while it is not reported yet for extraction of lipids from other microorganisms [265,305]. An expeller press is operating by mechanically crushing the biomass in an oil press [306]. Bead beating is based on a grinding mechanism [307,308], in which shaking vessels are filled with an agitated bead which breaks the cells by shaking in the vessel [309]. Bead milling is applicable for all types of oleaginous microorganisms and it has been applied to extract the lipids from oleaginous microalgae [263], bacteria [302], yeast [310] and fungi [311]. On the downside, although methods such as expeller press can increase the extraction efficiency of lipids, the increased content of pigments in the extract increases the overall cost of the downstream process [312]. Moreover, the application of such methods works only with low-moisture content samples, and the required drying of samples will increase cost and energy demands [306,312].

Another mechanical method is the microwave-assisted pretreatment of biomass that reduces the cost associated with the dewatering of algal biomass [283,313,314]. The major advantages of this method are the low energy input together with rapid treatment, high yield, purity of product, and avoidance of the use of hazardous substances [283,315]. However, the maintenance costs are considered the main limiting factor for the commercialization of the process [316]. Osmotic shock is another promising method, in which, by varying the salt concentration, hypo- and hyper-osmotic conditions are created, with the hypo-osmotic conditions playing a significant role in lipid removal from microorganisms. This is based on the fact that the high intracellular concentration of salt is balanced by water or fluids moving intracellularly, causing the cells to swell and burst [314,317,318]. Application of osmotic shock along with a mixture of polar and non-polar solvents for the extraction of lipids from wet *Chlamydomonas reinhardtii* cells resulted in an increase in lipid recovery by two times compared to other processes [317,319]. NaCl-induced osmotic stress was provided to *Chlorella vulgaris* for lipid production and it was suggested that NaCl-induced osmotic stress inhibits cell growth and improves lipid production as 30.1% higher lipid yield was obtained with stress compared to the control [320]. However, this method is affected by the cell wall properties and is species-specific, which makes it more complicated and, despite being a very simple method, its commercial application is very limited [298,321,322,323]. Furthermore, some authors used oxidative agents to improve the lipid extraction from oleaginous microorganisms. For example, Bai et al. (2014) used free nitrous acid (FNA) as an oxidative agent to treat microalgal cells and showed that FNA can increase the lipid extraction yield by 2.4-fold [324]. Electroporation is another technique that has been applied for lipid recovery from microalgal cells [314,325,326]. It has been reported that electroporation can result in increased lipid recovery, while it does not affect the composition and quality of lipids; however, further studies are necessary to prove that it is an efficient method for lipid recovery from oleaginous microorganisms [285,304,327]. Ultrasound-assisted extraction is another method that offers several benefits, such as being simple, eco-friendly, and time-efficient, with mild operation conditions and no need for chemicals [309,328,329,330,331,332,333]. However, the effect on the quality of the extracted lipids may be detrimental, as prolonged use of ultrasonication can produce free radicals [322].

Apart from mechanical and physical methods to facilitate the extraction of lipids from oleaginous microorganisms, biological methods have also been tested by several researchers. For example, a recombinant β-1,3-glucomannanase (plMAN5C) was tested for the degradation of the cell wall of microwave-pretreated cells of *Rhodosporidium toruloides* Y4 [334]. When it comes to algae cells, algaenan, a resistant, insoluble non-hydrolyzable biopolymer of the cell walls, makes the cell wall disruption of algae challenging. Cell wall disruption methods based on the use of different cocktails of enzymes have been tested. Enzymes such as neutral protease, papain, alkaline protease, cellulase, and lysozyme are used for this purpose, permitting easier lipid recovery by degrading cell wall polymers [304,335]. Although enzymatic degradation is poorly studied for the extraction of lipids from oleaginous microorganisms, the selectivity of reactions and minimal damage to the target product lead to an excellent amount of lipid recovery [336]. According to Fu et al. (2010), enzymatic disruption of the cell wall of *Chlorella* resulted in an approximate 14% increase in lipid extraction efficiency compared to unhydrolyzed microalgae [337]. Similarly, an almost 1.73-fold increment in lipid extraction was obtained with enzymatic hydrolysis of *C. vulgaris* in comparison with unhydrolyzed cultures [338]. The main advantages of the use of enzymes are their high selectivity, mild operating pressure–temperature conditions, and no corrosion compared to the use of physical or chemical methods of lipid extraction [304]. On the downside, the high cost associated with the use of enzymes and the long treatment time involved in the enzymatic pretreatment methods are the major drawbacks for large-scale application [339].

Finally, another method to lyse the cell walls is the use of antibiotics. Antibiotics are usually used to restrict the growth of bacteria; however, it can be used for lysing the growing Gram-negative bacteria at a lab scale. A very common group of antibiotics is β-lactam that interfere with peptidoglycan synthesis in Gram-negative bacteria, rendering the cells unable to maintain their osmotic pressure, with a subsequent release of all intracellular materials after disrupting the cell wall [302,340,341]. One significant drawback related to microbial oil production is related to the energy and solvent requirements for efficient lipid extraction from the cells, which often limits its commercial application. Hence, in addition to engineering microorganisms for increased lipid accumulation and desirable lipid composition, microorganisms have also been subjected to metabolic engineering with the purpose of attaining easier lipid recovery [112,342,343,344]. To avoid tremendous extraction processes, the engineering of strains capable of transporting the lipids extracellularly has also been proposed. One approach is to direct the flux toward the biofuel precursor free fatty acids, which can easily be transported out of the cells. To this end, *Y. Lipolytica* and *S. cerevisiae* have been engineered to excrete higher amounts of extracellular free fatty acids [345,346]. The same approach was used with *Escherichia coli* cells in which the synthetic pathway for fatty acids was coupled with an ABC transporter (such as MsbA, CydC, or putative ABC) to facilitate the excretion of the biofuel precursors into the medium, making their recovery easier [342]. This model test system has been postulated as a “plug-and-play” secreting system that can be used in various microorganisms such as yeasts [112,342,343].

### 7.3. Transesterification

The downstream process of single-cell oil production consists of four steps. When the aim is biodiesel production, the next step after the oil extraction is the transesterification procedure. During transesterification, the triacylglycerides that are recovered during the extraction are converted to fatty acid alkyl esters or fatty acid methyl esters (FAME) and glycerol as byproducts in the presence of ethanol or methanol and a catalyst [331]. The effectiveness of the transesterification reaction can be related to many different parameters. Some of them are the lipid origin (the type of microorganism), the reaction temperature, the selected solvents, the reaction time, and the type and content of the catalyst. Depending on these parameters, the reaction can be classified into different categories. In the present paragraph, the main transesterification methods are analyzed.

The transesterification methods for SCOs can be separated into conventional and direct. In the first category (conventional method), the downstream process has separate steps (referred to in the previous paragraphs) [347]. In the second category (direct or in-situ transesterification), the multiple steps are eliminated, as, with most of the cases, the lipid extraction and transesterification are achieved in one step [347]. In addition, depending on whether the catalysts occupy the same phase or not with the reaction mixture, transesterification can be characterized as homogenous or heterogeneous. Finally, based on the type of catalyst, the process can be acid, alkali, or enzymatic [347]. The transesterification process can be homogenous acid or alkali, heterogeneous acid, alkali, or enzymatic.

The catalysts are necessary for the increase of the reaction rate and the conversion yield. To improve the transesterification efficiency, change of catalyst and/or reaction conditions are necessary. The selection of the appropriate catalysts for each reaction constitutes a crucial step. In homogeneous catalysis, the catalyst has action in the same phase with the whole reaction mixture. The main problem with this type of transesterification is the possibility of soap formation because of the existence of free fatty acids [348]. Especially in microalgal cells, the use of alkali catalysts leads to soap formation that affects the downstream process. As such, acid and, more specifically, inorganic acids are recommended [349]. Depending on the type of catalyst, sometimes a bigger amount of catalyst is necessary, the reaction yield is decreased, and the cost is increased, something that makes homogeneous catalysts not ideal for industrial-scale use [348]. However, in heterogeneous catalysis, the catalyst appears in a different phase than that of the reaction mixture. So, as the catalyst can be separated from the total mixture and be reused, it appears selective, it needs less units, and it has a reduced cost [348,350], something that gives it a great advantage against homogeneous catalysts. This type of catalysis takes a longer time and needs higher temperatures and pressure [347].

The transesterification reaction is accelerated by catalysts, which are considered to be the main cost of this step and about 30%–40% of the total downstream cost [351]. In many studies and for many years, the chemical (acid or alkali) catalyzed transesterification has been reported as a better option and has been adopted by industries for biodiesel production. This method provides high conversion rates in less time. On the contrary, it is energy costly and not environment friendly. Consequently, enzymatic catalysts have gained ground [352].

The main difference between enzymatic and conventional transesterification is the ability to use biocatalysts in comparison with chemicals, while the above problem related to soap formation is avoided [353]. The main disadvantage of this method is the high cost that makes it unsuitable for use on an industrial scale. As the enzymes can act in a free form, their handling can be a difficult process, something that also increases the cost. This problem is partially solved with the use of immobilized enzymes, where the enzymes are immobilized on a carrier or a support material. In this case, the catalysts are stable and can be recovered and reused for more than one reaction cycle, while they have fewer byproducts [351,354,355,356]. Immobilization can be achieved in many ways, depending on the selected enzyme, the environment of the reaction, and the used solvents [355]. Moreover, the selection of the suitable enzyme and the determination of the reaction parameters, such as pH, temperature, concentration, time, play crucial roles in the whole process [356].

The most commonly used enzymes are from the lipase family. These consist of hydrolytic enzymes with the ability to catalyze the transesterification of fatty acids. Moreover, they can act in many environmental conditions, under a plethora of solvents, and they can be immobilized. Some lipases are produced by waste products and have high activity. So, they are promising enzymes that can increase the transesterification reaction efficiency, while contributing to a decrease in costs [352,355,356,357]. In addition, the enzymatic transesterification provides enhanced substrate specificity and also both catalytic stability and activity under room temperature conditions [352,358]. Moreover, the enzyme, except for its catalytic action, also contributes to the separation and recovery of the reaction products [352,355]. Thereafter, the use of enzyme catalysts will consist of a sustainable and eco-friendly transesterification method [359]. Among the existing catalysts, the most recent development with great prospect is the use of nanomaterials as catalysts in the transesterification process. It is refereed that this catalytic system overcomes some of the problems of heterogeneous catalysts, such as the prolonged reaction time, the enzyme deactivation, and the mass transfer limitations. In addition, nano-catalysts offer high specific areas and high catalytic activity, and the catalyst and the substrate appear to have enhanced interaction, and they increase the efficiency of the reaction [350,360]. Depending on the specific characteristics of every transesterification reaction, the synthesis of nanomaterials with specific characteristics is the next step. For instance, in some cases, the presence of metal in nanomaterials constitutes an advantage for the reaction, while in thermally induced reactions, a porous nanomaterial with catalytic properties seems to be necessary [350,361]. The major challenge and research aim of this catalytic method is the finding of green methods for nanomaterial synthesis. These materials will be able to replace the acid and base catalysts, obeying a whole biorefinery concept [350]. In some cases, these techniques are completed with additional methods, leading to an enhanced extractive–transesterification process. For instance, the microwave method can be enhanced with ultrasounds, which increase process efficiency by reducing costs, making them ideal for large-scale productions [362].

Finally, two widely used direct transesterification methods are these of supercritical and microwave-assisted conditions. The special characteristics of the first method are the production of highly purified products and the reduced energy cost, while the second one appears to have higher efficiency on yield and time [363]. In the supercritical method, the water of the wet microalgal biomass acts as co-solvent to the solvent (the most commonly used solvents are methanol and ethanol), and the de-watering step is not necessary. In the microwave method, the microwaves can penetrate and destroy the walls of dry microalgal cells. So, the extraction and transesterification of lipids are achieved in one step [363,364,365]. In the case of supercritical transesterification, the selection of a more eco-friendly solvent, produced from renewable sources, can lead to a more environmentally sustainable method [365,366]. Both methods are environment friendly as they reduce the use of chemicals and energy, limiting the partial steps with the single-step extraction–transesterification process and, as a result, decrease the cost of the whole downstream process.

So, there are many ways towards the achievement of a successful transesterification reaction. In most of the cases, the combination of two or more methods leads to better results. Biodiesel production must basically deal with the cost of the individual steps of the downstream process and their environmental issues. The selection of the appropriate method should lead to the production of high-quality biodiesel, preferably in a way that restricts the cost and lessen the environmental footprint. Something like that could be done with the application of the biorefinery concept, through the selection of specific microorganisms, and with the use of processes that do not at all need or minimize the use of toxic chemicals. The development of transesterification catalysts with the desirable characteristics, which cover the previous characteristics and can be used on an industrial scale, is a crucial and urgent requirement.

### 7.4. Purification of Biodiesel

A major concern about biodiesel production is final product quality. In the downstream processing of microalgal cell oils, after the transesterification step, the product does not yet have the required purity. The final mixture may contain soap, enzymes, metal ions, water, acid or base solvent, and non-desirable lipids that need to be separated [367]. The methods for this separation are not predefined, but they depend on the previous steps. For example, if the transesterification reaction is achieved on microalgal biomass, the first stage of the purification step is the separation of lipids from biomass, which can be done in most of the cases with filtration or centrifugation [347].

However, when biodiesel is the main product, glycerol constitutes the major byproduct that needs to be removed. As a result, the first step is the separation of polar glycerol from non-polar fatty acids, which can be achieved by gravitational or centrifugation techniques. After that, the fatty acids can be better clarified [347,368].

As biodiesel crude contains water, amounts of the used catalysts (e.g., enzymes, soluble, nanomaterials), glycerol (monoacyl-, diacyl-), triglycerides, and soap, more techniques are needed for its purification. Different methods have been reported for this step, such as the use of solvents like hexane in combination with vacuum, sodium sulfate, and filtration to remove the byproducts [347,368]. In most of the cases, the selected method depends on the previous steps and, as a result, what type of byproducts co-exist in the final mixture. Some of the most usual techniques towards this aim are dry-washing, wet-washing, and membrane separation [347]. Each method has sub-categories and a plethora of parameters that should be defined each time, and they are analyzed below.

Wet washing constitutes the most well-known, traditional, and conventional method for biodiesel purification. It is suitable to remove excess contaminants and chemicals from the previous steps. The major disadvantages of this method are the demand of high amounts of water and the need for absolute water removal by drying the final product, pointing out the wastewater need for extra treatment before its disposal. As a result, the need for extra techniques for water removal and disposal increases the production time and the total operation cost [274,369,370,371].

The dry-washing technique aims to replace the above one, and, as there is no need for water, there is no product loss, and it provides the extra advantage of selecting the most proper adsorbent. There are many efficient compounds for dry washing, such as silica and starch and cellulolytic derivatives, and ion exchange resin [372,373,374]. The search for more economical, eco-friendly materials, leading to biodiesel purification without interacting and changing the main product, seems to be necessary. Some materials with these characteristics have already been reported, such as chamotte, which has been proposed to be of low cost and high efficiency for ethyl biodiesel purification [375].

Except for the above two conventional methods, some novel methods are gaining attention. The most developed among them is the use of membrane technology [376]. The membranes are composed of support and coating materials, and the whole process is based on rejection coefficients. Each material has special characteristics, making it suitable for different processes. Membranes of poly vinylidene fluoride (PVDF) and poly dimethyl siloxane are two commonly used materials [347], together with ceramic materials that are suitable for organic solvents [369]. The most important characteristic of membranes is their chemical and thermal stability, so they can be used on the desirable solvents, pH, and temperatures, reducing the degradation and corrosion rates and the size of their pores [347,369,376,377]. It is a technology with lower operation costs but high purchase costs compared to previous ones, making it inappropriate for industrial-scale use. In addition, a basic drawback of membranes is the fouling problems. Soap, glycerol, solutes, and particles can accumulate on the pores, causing the process to stop. As a result, specific solvents should be used in order to overcome this problem [377,378]. On the contrary, the low energy consumption of the membranes operation reduces the environmental footprint, making it a method with future perspectives [376,377,378].

A recent research focused on the improving of these steps, using more eco-friendly solvents and sorbents and, on the integration of previous steps, for example, stimulating transesterification and purification. In addition, some scientists use a combination of the above techniques, creating, for instance, a two-stage process starting with wet- and continuing with dry-washing, leading better quality biodiesel [369].

Obviously, biodiesel as a final product has to be purified and meet the quality properties of organizations like the European Union and the American Society of Testing and Materials. For this reason, the above methods constitute the final step of the downstream process of biodiesel production. The purification process has to be balanced among the environmental, operation, and purchase costs, and the efficiency. The next research aim is to reduce of the economic and environmental cost of this step and as a result of the whole process, making biodiesel production more profitable with less of an environmental footprint.

### 7.5. Concentration of Omega-3 Fatty Acids

Single-cell oils, depending on their form, can be used both as a food supplement for the food and nutraceutical industries and as a renewable energy source. The most common used lipids for the first application are the omega-3 and omega-6 lipids. For the second application, a transesterification step is necessary for the production of fatty acid alkyl or methyl esters, commonly named biodiesel. In both cases, there are techniques that are able to clean the produced lipids, remove co-products, and purify the basic compounds so as to increase the quality and content of the final product. For the enrichment of the ω-3 and ω-6 lipids with the desired compounds and removal of co-products, the well-used methods are winterization, molecular distillation, and urea complexation.

The most common byproducts that have to be removed are the monoacyl-, diacylglycerols, and free fatty acids. Urea complexation and molecular distillation lead to quite a successful removal but increase the cost of the whole process. On the contrary, winterization has been proposed as an alternative method that dramatically increases the PUFA content, avoiding in parallel high temperatures that are aggravating on products for nutraceutical use [379]. In general, in this method, oils are treated with organic solvents under low temperatures (0, –20, –80 °C) for some cycles, leading to crystallization of some compounds. The solvent is selected to separate the polyunsaturated fatty acids from the other compounds (saturated fatty acids) based on their solubility and their melting point [380]. This process creates oil fragments that can lead to a doubling of ω-3 content [379]. Winterization can be used in combination with urea complexation, increasing the content of PUFAs and reducing the content of FFAs, resulting in up to 95% DHA purity [172]. In urea complexation, saturated and less unsaturated FFAs are separated from polyunsaturated FAs, with the help of crystalline urea, which is used as the compound to which the FAs can be connected [381,382]. Urea creates crystals with FFAs, and, in turn, PUFAs are finally relatively clear at the final mixture. In some studies, in combination with enzymatic or chemical hydrolysis, urea complexation leads to a total reduction of FFAs, especially monounsaturated fatty acids [379,381]. In any case, parameters like the ratio between urea and fatty acids and the time of the crystallization play important roles in the process [383,384]. A study on extraction and purification of DHA concluded that urea complexation with the correct parameters increased the DHA purity from 30% to 60% [383]. Also, urea complexation can be used in combination with molecular distillation, obtaining highly concentrated PUFAs [385]. In this method, lipids are separated based on their molecular weight. The whole process is achieved under vacuum, so there is a need for stable compounds and substrates [380,386].

Consequently, these methods are appropriate, alone or in combination, to enhance the quantity and quality of ω-3 and ω-6 lipids. In each case, based on the available conditions, microorganism, and the desired compound that needs to be separated, scientists should find the appropriate method with the most effective parameters so as to achieve the optimal result.

## 8. Conclusions

The major challenge faced by biodiesel industries is the availability of low-cost feedstocks. Utilization of refined vegetable oils for the production of biofuels increases the total cost of production, whereas it also creates concern on the food vs. fuel debate. Hence, in order to reduce the high cost involved in feedstock and the social impact, microbial oil can be a plausible alternative resource for food and fuel applications. However, high costs associated with growth media for the cultivation of oleaginous microorganisms again raised a similar concern, but the utilization of renewable carbon sources mostly obtained from waste streams can solve this problem. Further, the integration of biofuels production from oleaginous microorganisms with various value-added products help to reduce the overall production cost. Likewise, getting omega-3 fatty acids from diminishing fish stock creates long term problems for the aquatic ecosystem. Oleaginous thraustochytrids and certain microalgae have the capability to replace fish feedstock for PUFA production in a sustainable way.

## Figures and Tables

**Table 1:** A list of oleaginous microorganisms cultivated on various sources and their lipid content.

Oleaginous Microorganisms	Substrates	Lipid Content (%, *w/w*)	References
**Microalgae**			
*Scenedesmus* sp	Photoautotrophic (modified Chu 13 medium) + bubbled with simulated biogas (CO2:CH4 40:60)	34.10	[113]
*Chlorella protothecoides*	Glucose	49	[114]
*Tetraselmis elliptica*	Photoautotrophic (Flory medium)	14	[115]
*C. vulgaris NIES-227*	Heterotrophic cultivation on glucose under nitrogen limitation	89	[116]
*Auxenochlorella protothecoides*	Organosolv pretreated birch biomass hydrolysates	66	[71]
*Auxenochlorella protothecoides*	Organosolv pretreated spruce biomass hydrolysates	63	[71]
*Botryococcus braunii*	Photoautotrophic (modified Chu 13 medium)	28	[117]
*Chlamydomonas reinhardtii, CC1010*	Photoheterotrophic (TAPN^-^ + 0.1% glucose)	59	[118]
**Yeast and filamentous fungi**			
*Cryptococcus* sp. (KCTC 27583)	Pretreated banana peel	34	[119]
*Rhodosporidium kratochvilovae* HIMPA1	Cassia fistula L. fruit pulp	53.18	[42]
	Hemp seed aqueous extract	55.56	[44]
	Phenol 1 g/L + Glucose (7%)	64.92	[120]
	Hydrophobic waste (clarified butter sediment waste medium	70.74	[7]
*Trichosporon fermentans* CICC 1368	pre-treated waste sweet potato vines under simultaneous saccharification and fermentation (SSF)	36	[121]
*Rhodosporidium toruloides*	Brewers’ spent grain	56	[45]
*Lipomyces starkeyi*	Xylose and glucose	48	[122]
*Rhodotorula glutinis*	Monosodium glutamate with glucose	20	[123]
*Cryptococcus curvatus*	Waste cooking oil	70	[124]
	Glucose	53	
*Lipomyces starkeyi* CBS 1807	Sweet sorghum stalks juice	30	[82]
*Fusarium oxysporum*	Sweet sorghum stalks (12% *w/w* solid load)	22	[125]
	Glucose	42	
	Fructose	26	
	Sucrose	49	
	Glucose, fructose and sucrose mixture	53	
*Fusarium equiseti* UMN-1	Glucose	56	[126]
*Sarocladium kiliense* ADH17	Glucose and glycerol	33	[127]
*Mortierella alpina* LP M 301	Glucose with potassium nitrate	31	[103]
*Microsphaeropsis* sp.	Corncob waste liquor	22	[96]
**Bacteria**			
*Rhodococcus opacus* DSM 1069	Ethanol organosolv lignin	4	[111]
*R. opacus* PD630	Dairy wastewater	14	[128]
	Dextrose	70	
*R. opacus* DSM 43205	Biomass gasification wastewater	66	[129]
*Gordonia sp. DG*	Olive oil	13	[130]
	Sesame oil	50	
	Cotton oil	50	
	Pea-nut oil	40	
	Maize oil	40	
	Sunflower oil	52	
*R. opacus* PD630	Kraft hardwood pulp	46	[131]

**Table 2:** A list of oleaginous microorganisms with their EPA and DHA content.

Oleaginous Microorganisms	Substrate	DHA Concentration (%, Total Lipid)	EPA Concentration (%, Total Lipid)	References
**Thraustochytrids**				
*Aurantiochytrium* sp. ATCC PRA-276	Glucose (30 g/L)	5.5	-	[185]
		12.5	-	
*Aurantiochytrium* sp. KRS101	Orange peel extract glucose (5.9 g/L), fructose (5.6 g/L), organic acids	14.31	-	[186]
	5 g/L glucose + orange peel extract glucose (5.9 g/L), fructose (5.6 g/L), organic acids	14.18	-	
*Aurantiochytrium* sp. KRS101	Modified basal medium glucose (60 g/L)	19.88	-	[187]
*Schizochytrium limacinum* SR 21	Glucose (90 g/L)	14.72	-	[188]
	Glycerol (100 g/L)	18.38	-	
*Aurantiochytrium* 4W-1b	Glucose (30 g/L)	27.9	-	[189]
*Aurantiochytrium* SW1	Fructose (70 g/L)	25	-	[190]
*Aurantiochytrium* sp. YLH70	High-fructose corn syrup	46.3	-	[191]
*Schizochytrium limacinum* SR21	Organosolv-pretreated spruce hydrolysate (60 g/L glucose)	66.72	-	[192]
*Aurantiochytrium* sp. ATCC PRA-276	Organosolv-pretreated birch hydrolysate (30 g/L glucose)	35.76	-	[23]
**Microalgae**				
*P. tricornutum*	Autotrophic	1.65	13.43	[21]
	Mixotrophic, Glucose (2 g/L)	3.56	18.38	
	Mixotrophic, Birch hydrolysates	4.32	19.80	
	Mixotrophic, Spruce hydrolysates	4.89	19.87	
*Chlorella minutissima* UTEX 2341	Photoautotrophic	-	31.8	[193]
*Nannochloropsis salina*	Photoautotrophic	-	28	[194]
*Nannochloropsis* sp.	Photoautotrophic		25	[195]
*Alexandrium sanguinea*	Photoautotrophic	23.8	20.1	[196]
*Chlorella ellipsoidea*	Photoautotrophic	3.1	34.6	
*Chlamydomonas* sp	Photoautotrophic	3.2	19.7	
*Crypthecodinium cohnii* ATCC 30772	Glucose	-	43.6	[197]
*C. cohnii* CCMP 316	Glucose + n-Dodecane	51	-	[171]
**Filamentous fungi**				
*Mortierella alpina* ST1358*	GY medium (2% (*w/v*) glucose and 1% yeast extract)	-	26.4	[198]
*Mortierella alliacea* YN-15	-	-	1.3 -13	[199]
*Pythium irregulare*	Sweet whey permeate	-	25.2	[200]
**Filamentous fungi for γ-linolenic acid** (**GLA**) **production**				
*Mortierella isabellina*	Glucose	GLA/oil 3.5%		[201]
*Cunninghamella echinulata*	Xylose (C/N 285)	GLA/oil 22.0%		[99]
*M. isabellina* ATHUM 2935	Xylose (C/N 235)	GLA/oil 8.4%		
*C. echinulata*		GLA/oil 9–16%		[202]
*M. isabellina*		GLA/oil 1.5–4.5%		
*M. isabellina*	Gluocse	GLA/oil 3.4%		[203]
*M. isabellina*	Pectin	GLA/oil 6.1%		
*C. echinulata*	Glucose	GLA/oil 16.5%		
*C. echinulata*	Starch	GLA/oil 14.2%		
*C. echinulata*	Tomato waste hydrolysate (TWH)	GLA/oil 11.7%		[204]
*C. echinulata*	Glucose	GLA/oil 19.7%		[19]
